# Supplementary material for: Altered brain network topology in children with auditory processing disorder: A resting-state multi-echo fMRI study
Source: Neuroimage Clin. 2022 Aug 1;35:103139. doi: 10.1016/j.nicl.2022.103139 (PMC9421544; doi:10.1016/j.nicl.2022.103139)
Supplement: Supplementary data 1 [file mmc1.docx]

1. Selection of the denoising pipeline

**Figure S1** Results from benchmarking the denoising pipelines. Quality control functional connectivity (QC-FC) measures were applied as recommended by [Parkes and colleagues (2018)](https://paperpile.com/c/JO47eU/IAzl) on seven different commonly used denoising procedures to quantify the efficacy (A, B; QC-FC correlation, QC-FC distance dependence) and efficiency (C; loss of temporal degree of freedom; tDOF-loss) of denoising pipelines. These pipelines were assessed based on the combination of different regressors and their temporal derivatives, including head motion parameters (HMP), component-based noise correction method [(](https://paperpile.com/c/JO47eU/d1N4+E05h)aCompCor; [Behzadi et al., 2007; Muschelli et al., 2014)](https://paperpile.com/c/JO47eU/d1N4+E05h), independent component analysis strategy for automatic removal of motion artifacts [(](https://paperpile.com/c/JO47eU/7aGL)ICA-AROMA; [Pruim et al., 2015)](https://paperpile.com/c/JO47eU/7aGL) and two physiological signals (Phys) artifacts such as white matter signal (WM) and global signal regression (GSR). Based on the results, the combination of ICA-AROMA+8Phys+4GSR had the best performance compared to other pipelines. See [Parkes et al. (2018) and Ciric et al. (2017)](https://paperpile.com/c/JO47eU/IAzl+tZcf) for a full overview of the measure and concepts used in this benchmarking procedure.

2. Head motion profile

**Table S1**

*Head motion profile of participants*

***Note:*** FD - framewise displacement, Min - minimum, Max - maximum, var - variance, DVARS - D is referring to the temporal derivative of time series and VARS is referring to RMS variance over voxel.

**Figure S2** The distribution of mean FD across APD and HC groups. FD - framewise displacement, HC - healthy control, APD - auditory processing disorder.

3. Whole-brain averaged network measures

**Figure S3** Results from the average global network integration measure (global efficiency, characteristic path length), network segregation (mean local efficiency and clustering coefficient, modularity optimization), and small-worldness metric. The 95% confidence interval between APD (red line) and HC (blue line) groups was also calculated.

4. LiSN-S data

**Table S2**

*LiSN-S data dispersion for all the participants*

***Note:*** There are multiple modes, but the smallest value is shown. The data shown here are only on 54 participants with three missing values in APD(n=2) and HC groups (n=1). SD - Standard deviation, Adv - advantage, HC - healthy control, APD - auditory processing disorder.

5. Hub analysis

**Figure S4** Changes in modular architecture from 10% to 40% network densities. (A): APD group. (B): HC group.

**Table S3**

*Brain hubs and their roles in APD and HC groups*

***Note:*** The difference in hub role is shown for both groups. Nodes that are not assigned C or P are identified as non-hub. Normalized participation coefficient (PC_norm_), within-module z-score degree (WMZ), connector hub (C), provincial hub (P).

**Table S4**

*Results from group differences between HC and APD groups with and without age as a regressor*

***Note:***Age represents results based on age as a nuisance covariate. FDR - false discovery rate, Bon - Bonferroni correction, *p -* p value, T - test statistic, Hem - hemisphere, ROI - region of interest, Centroid - ROI’s coordinate in standard space, TempOcc – temporo-occipital cortex, TempPar - temporal-parietal network, Ins - intraparietal sulcus, DMN - default mode network, SalVentAttn - salience ventral attention network, Cont - control network, DorsAttn - dorsal attention network, L - left hemisphere, R - right hemisphere.

6. Comparisons between Gordon and Schaefer parcellations in PC measure

**Figure S5** Test statistical map of the brain region in PC measure. The test statistic map was derived from the group analysis of PC measure based on Gordon (333 ROIs) and Schaefer (300 ROIs) parcellations. Colors represent statistical scores for each ROI and are coded based on their negative or positive values. Regions with smaller t values are coded as blue and regions with greater t values are colored yellow. PC - participation coefficient, L - left hemisphere, R - right hemisphere, t-stat - test statistic.

**Table S5**

*Overlapping ROIs between Schaefer and Gordon parcellations based on PC measure*

***Note:*** FDR - false discovery rate, p - p value, T - test statistic value, Hem - hemisphere, ROI - region of interest, coordinate - ROI’s coordinate in standard space, TempOcc - temporalOccipital cortex, TempPar - temporal-parietal network, Ins - insula, IPS - intraparietal sulcus, DMN - default mode network, SalVentAttn - salience ventral attention network, Cont - control network, DorsAttn - dorsal attention network, pCun PCC - precuneus/posterior cingulate cortex, VisCen - visual central, L - left hemisphere, R - right hemisphere.

7. Meta-analytic correlation

**Table S6**

*Meta-analytic correlation between cognitive terms and significant ROIs obtained from Gordon and Schaefer parcellation*

***Note:*** The top 20 associated cognitive terms are shown for each significant ROI according to Gordon and Schaefer parcellations. The meta-analytic terms were derived from the Neurosynth database [(Yarkoni et al., 2011)](https://paperpile.com/c/JO47eU/r2EZ). Cognitive terms were selected based on their meta-analytic coactivation scores (Pearson correlation; uncorrected).

# References

Behzadi, Y., Restom, K., Liau, J., & Liu, T. T. (2007). A component-based noise correction method (CompCor) for BOLD and perfusion-based fMRI. Neuroimage, 37(1), 90-101. <https://doi.org/10.1016/j.neuroimage.2007.04.042>

Ciric, R., Rosen, A. F., Erus, G., Cieslak, M., Adebimpe, A., Cook, P. A., ... & Satterthwaite, T. D. (2018). Mitigating head motion artifact in functional connectivity MRI. *Nature protocols*, *13*(12), 2801-2826. <https://doi.org/10.1038/s41596-018-0065-y>

Muschelli, J., Nebel, M. B., Caffo, B. S., Barber, A. D., Pekar, J. J., & Mostofsky, S. H. (2014). Reduction of motion-related artifacts in resting-state fMRI using aCompCor. Neuroimage, 96, 22-35. <https://doi.org/10.1016/j.neuroimage.2014.03.028>

Parkes, L., Fulcher, B., Yücel, M., & Fornito, A. (2018). An evaluation of the efficacy, reliability, and sensitivity of motion correction strategies for resting-state functional MRI. *Neuroimage*, *171*, 415-436. <https://doi.org/10.1016/j.neuroimage.2017.12.073>

Pruim, R. H., Mennes, M., van Rooij, D., Llera, A., Buitelaar, J. K., & Beckmann, C. F. (2015). ICA-AROMA: A robust ICA-based strategy for removing motion artifacts from fMRI data. *Neuroimage*, *112*, 267-277. <https://doi.org/10.1016/j.neuroimage.2015.02.064>

Yarkoni, T., Poldrack, R. A., Nichols, T. E., Van Essen, D. C., & Wager, T. D. (2011). Large-scale automated synthesis of human functional neuroimaging data. *Nature methods*, *8*(8), 665-670. <https://doi.org/10.1038/nmeth.1635>
