# Supplementary material for: Altered brain network topology in children with auditory processing disorder: A resting-state multi-echo fMRI study
Source: Neuroimage Clin. 2022 Aug 1;35:103139. doi: 10.1016/j.nicl.2022.103139 (PMC9421544; doi:10.1016/j.nicl.2022.103139)
Supplement: Supplementary data 2 [file mmc2.docx]

**Table S1**

*Head motion profile of participants*

| **Group** | **FD max** | **FD mean** | **FD var** | **DVARS max** | **DVARS mean** | **DVARS var** |
| --- | --- | --- | --- | --- | --- | --- |
| APD1 | 0.43 | 0.07 | 0.05 | 61.32 | 31 | 4.1 |
| APD2 | 1.58 | 0.21 | 0.24 | 73.27 | 37.31 | 8.53 |
| APD3 | 0.95 | 0.13 | 0.09 | 48.4 | 30.59 | 3.99 |
| APD4 | 0.593 | 0.06 | 0.05 | 43.44 | 29.26 | 2.19 |
| APD5 | 0.48 | 0.11 | 0.07 | 56.8 | 33.48 | 3.6 |
| APD6 | 0.369 | 0.11 | 0.064 | 35.772 | 27.745 | 3.025 |
| APD7 | 0.303 | 0.07 | 0.05 | 33.16 | 23.729 | 2.229 |
| APD8 | 0.373 | 0.09 | 0.048 | 36.657 | 25.958 | 2.794 |
| APD9 | 1.81 | 0.11 | 0.234 | 69.187 | 32.863 | 7.209 |
| APD10 | 0.709 | 0.1 | 0.099 | 66.001 | 30.787 | 4.231 |
| APD11 | 1.51 | 0.09 | 0.147 | 73.827 | 31.043 | 6.282 |
| APD12 | 0.22 | 0.05 | 0.035 | 37.511 | 24.36 | 1.627 |
| APD13 | 1.607 | 0.07 | 0.148 | 91.006 | 27.155 | 7.923 |
| APD14 | 0.65 | 0.08 | 0.069 | 45.885 | 27.869 | 3.328 |
| APD15 | 1.427 | 0.13 | 0.15 | 60.881 | 31.753 | 5.777 |
| APD16 | 1.329 | 0.16 | 0.154 | 62.269 | 35.179 | 5.222 |
| APD17 | 0.771 | 0.13 | 0.115 | 52.933 | 31.172 | 4.68 |
| APD18 | 0.299 | 0.1 | 0.048 | 44.915 | 38.538 | 2.512 |
| APD19 | 0.546 | 0.07 | 0.054 | 47.195 | 35.277 | 2.332 |
| APD20 | 0.481 | 0.09 | 0.072 | 42.204 | 28.637 | 2.89 |
| APD21 | 0.829 | 0.11 | 0.115 | 40.121 | 27.122 | 3.232 |
| APD22 | 1.55 | 0.11 | 0.149 | 87.77 | 29.363 | 6.742 |
| APD23 | 0.435 | 0.09 | 0.069 | 39.063 | 30.322 | 2.268 |
| APD24 | 0.946 | 0.13 | 0.153 | 59.271 | 34.877 | 5.476 |
| APD25 | 1.082 | 0.1 | 0.154 | 52.384 | 32.785 | 4.451 |
| APD26 | 0.335 | 0.09 | 0.054 | 38.12 | 32.036 | 2.404 |
| APD27 | 1.049 | 0.16 | 0.152 | 68.94 | 29.69 | 5.894 |
| APD28 | 1.087 | 0.1 | 0.157 | 82.852 | 31.89 | 6.929 |
| HC1 | 1.08 | 0.07 | 0.09 | 53.62 | 33.39 | 2.22 |
| HC2 | 0.18 | 0.06 | 0.03 | 31.11 | 27.85 | 1.4 |
| HC3 | 1.04 | 0.11 | 0.12 | 52.83 | 35.28 | 4.53 |
| HC4 | 0.28 | 0.07 | 0.04 | 41.55 | 27.1 | 3.53 |
| HC5 | 0.53 | 0.12 | 0.09 | 45.6 | 32.33 | 4.17 |
| HC6 | 0.7 | 0.09 | 0.07 | 40.86 | 27.32 | 3.08 |
| HC7 | 0.68 | 0.12 | 0.1 | 46.62 | 32.95 | 3.47 |
| HC8 | 0.634 | 0.1 | 0.07 | 45.88 | 31.91 | 3.71 |
| HC9 | 0.66 | 0.15 | 0.11 | 46.68 | 37.9 | 2.34 |
| HC10 | 0.22 | 0.06 | 0.03 | 40.13 | 31.15 | 1.71 |
| HC11 | 0.52 | 0.11 | 0.07 | 39.95 | 32.19 | 2.42 |
| HC12 | 0.43 | 0.12 | 0.09 | 41.71 | 34.48 | 2.66 |
| HC13 | 0.734 | 0.15 | 0.1 | 48.14 | 29.89 | 2.9 |
| HC14 | 1.506 | 0.2 | 0.24 | 85.68 | 33.37 | 10.2 |
| HC15 | 0.14 | 0.05 | 0.02 | 33.94 | 27.28 | 2.59 |
| HC16 | 0.984 | 0.18 | 0.134 | 47.72 | 34.708 | 3.008 |
| HC17 | 0.394 | 0.1 | 0.061 | 34.757 | 30.38 | 1.215 |
| HC18 | 1.628 | 0.18 | 0.219 | 107.443 | 42.327 | 10.371 |
| HC19 | 0.184 | 0.05 | 0.029 | 38.019 | 29.106 | 2.772 |
| HC20 | 0.446 | 0.11 | 0.073 | 43.59 | 27.45 | 2.721 |
| HC21 | 1.667 | 0.19 | 0.277 | 117.48 | 35.817 | 11.14 |
| HC22 | 0.57 | 0.15 | 0.101 | 37.94 | 31.402 | 1.776 |
| HC23 | 0.479 | 0.1 | 0.065 | 37.986 | 26.953 | 1.905 |
| HC24 | 0.834 | 0.17 | 0.111 | 44.749 | 31.848 | 2.549 |
| HC25 | 1.094 | 0.12 | 0.146 | 87.613 | 33.708 | 7.281 |
| HC26 | 3.297 | 0.2 | 0.366 | 118.555 | 34.156 | 13.765 |
| HC27 | 0.186 | 0.06 | 0.028 | 34.233 | 25.439 | 3.001 |
| HC28 | 0.334 | 0.08 | 0.051 | 37.674 | 30.572 | 2.504 |
| HC29 | 0.277 | 0.06 | 0.033 | 33.038 | 27.823 | 1.951 |

***Note:*** FD - framewise displacement, Min - minimum, Max - maximum, var - variance, DVARS - D is referring to the temporal derivative of time series and VARS is referring to RMS variance over voxel.
