# Supplementary material for: Altered brain network topology in children with auditory processing disorder: A resting-state multi-echo fMRI study
Source: Neuroimage Clin. 2022 Aug 1;35:103139. doi: 10.1016/j.nicl.2022.103139 (PMC9421544; doi:10.1016/j.nicl.2022.103139)
Supplement: Supplementary data 3 [file mmc3.docx]

**Table S2**

*LiSN-S data dispersion for both APD and HC groups*

|  | **Low cue** | **High cue** | **Talker adv** | **Spatial adv** | **Total adv** |
| --- | --- | --- | --- | --- | --- |
| **Mean** | -0.1333 | 0.3852 | -0.5000 | 0.0056 | 0.5074 |
| **Median** | -0.02000 | 0.5000 | -0.4000 | 0.0000 | 0.5500 |
| **Mode** | -0.30 | 0.50 | -0.40 | 0.00 | 0.90 |
| **Std. Deviation** | 1.06700 | 0.98716 | 0.92939 | 1.34563 | 1.00974 |
| **Variance** | 1.138 | 0.974 | 0.864 | 1.811 | 1.020 |
| **Range** | 5.20 | 3.90 | 4.20 | 6.00 | 3.90 |
| **Minimum** | -2.70 | -1.90 | -2.60 | -3.70 | -1.40 |
| **Maximum** | 2.50 | 2.00 | 1.60 | 2.30 | 2.50 |

***Note:*** There are multiple modes, but the smallest value is shown. The data shown here are based only on 54 participants with three missing values in APD(n=2) and HC groups (n=1). SD - Standard deviation, Adv - advantage, HC - healthy control, APD - auditory processing disorder.
