# Supplementary material for: Altered brain network topology in children with auditory processing disorder: A resting-state multi-echo fMRI study
Source: Neuroimage Clin. 2022 Aug 1;35:103139. doi: 10.1016/j.nicl.2022.103139 (PMC9421544; doi:10.1016/j.nicl.2022.103139)
Supplement: Supplementary data 4 [file mmc4.docx]

**Table S3**

*Brain hubs and their roles in APD and HC groups*

| **ROIs** | **L/R** | **Regions** | **MNI** | | | **HC** | **PC_norm_** | **WMZ** | **APD** | **PC_norm_** | **WMZ** |
| --- | --- | --- | --- | --- | --- | --- | --- | --- | --- | --- | --- |
| 2 | L | SMhand | -19 | -49 | 65 |  | 0.516 | -0.061 | P | 0.421 | 1.140 |
| 5 | L | Visual | -18 | -86 | 21.6 | C | 0.537 | 1.273 | P | 0.442 | 1.213 |
| 6 | L | Default | -47 | -58 | 30.8 |  | 0.710 | 0.961 | C | 0.584 | 1.903 |
| 25 | L | Default | -6 | 42 | 35.1 | P | 0.475 | 1.856 |  | 0.560 | 0.541 |
| 27 | L | CinguloOperc | -8 | 15 | 33.8 |  | 0.520 | 0.459 | C | 0.504 | 1.037 |
| 36 | L | SMhand | -5 | -28 | 60.4 |  | 0.309 | 0.893 | P | 0.373 | 1.379 |
| 50 | L | SMhand | -29 | -45 | 61.7 |  | 0.485 | -1.135 | P | 0.344 | 1.046 |
| 52 | L | DorsalAttn | -43 | -45 | 43 | C | 0.546 | 2.512 | P | 0.486 | 1.946 |
| 57 | L | SMhand | -28 | -37 | 61.4 |  | 0.390 | 0.251 | P | 0.409 | 1.342 |
| 74 | L | DorsalAttn | -44 | 36 | 8.5 |  | 0.606 | -0.300 | P | 0.373 | 1.209 |
| 80 | L | VentralAttn | -29 | 21 | -14 | C | 0.518 | 1.508 |  | 0.602 | 0.042 |
| 82 | L | CinguloOperc | -37 | 8.9 | -0.9 | C | 0.578 | 1.042 |  | 0.557 | 0.840 |
| 87 | L | DorsalAttn | -20 | -65 | 51.4 | C | 0.567 | 1.502 |  | 0.755 | 0.571 |
| 100 | L | DorsalAttn | -46 | -58 | -7.9 | C | 0.581 | 1.466 |  | 0.649 | 0.680 |
| 103 | L | CinguloOperc | -55 | -32 | 23 | C | 0.510 | 1.224 | P | 0.498 | 1.112 |
| 111 | L | CinguloOperc | -52 | -1 | 5 | C | 0.508 | 1.915 | P | 0.477 | 1.676 |
| 126 | L | Default | -63 | -29 | -7.2 | C | 0.544 | 1.060 |  | 0.497 | 0.993 |
| 145 | L | Default | -16 | 49 | 37.2 | P | 0.475 | 1.751 |  | 0.497 | 0.943 |
| 146 | L | Default | -20 | 56 | 27.5 | C | 0.528 | 1.021 |  | 0.621 | 0.712 |
| 162 | R | Default | 12 | -52 | 34.5 |  | 0.845 | 0.968 | C | 0.740 | 1.601 |
| 163 | R | SMhand | 21 | -48 | 66.1 |  | 0.485 | -0.132 | P | 0.340 | 1.171 |
| 166 | R | Visual | 22 | -85 | 23.7 |  | 0.490 | 0.999 | P | 0.437 | 1.038 |
| 185 | R | CinguloOperc | 8.6 | 4.2 | 40.1 |  | 0.533 | 0.841 | C | 0.515 | 1.228 |
| 191 | R | SMhand | 4.8 | -27 | 64.8 |  | 0.262 | 0.943 | P | 0.379 | 1.447 |
| 195 | R | SMhand | 6.8 | -8 | 50.9 | C | 0.503 | 1.410 |  | 0.591 | 0.456 |
| 211 | R | DorsalAttn | 39 | -43 | 40.4 | P | 0.435 | 1.240 | C | 0.562 | 1.469 |
| 231 | R | VentralAttn | 49 | -27 | -0.1 |  | 0.634 | 0.520 | C | 0.638 | 1.055 |
| 245 | R | CinguloOperc | 40 | 10 | -1.6 | C | 0.635 | 1.295 |  | 0.625 | 0.830 |
| 251 | R | Visual | 27 | -55 | 54.2 | C | 0.645 | 1.094 |  | 0.754 | 0.931 |
| 252 | R | DorsalAttn | 23 | -66 | 51.8 | C | 0.595 | 1.060 |  | 0.734 | 0.429 |
| 253 | R | DorsalAttn | 32 | -64 | 33.8 | C | 0.557 | 1.049 |  | 0.637 | 0.473 |
| 269 | R | Auditory | 54 | -14 | 16.9 |  | 0.451 | 0.999 | P | 0.475 | 1.216 |
| 273 | R | FrontoParietal | 42 | 29 | 21.6 | C | 0.516 | 1.713 | P | 0.325 | 1.846 |
| 275 | R | DorsalAttn | 47 | 7.8 | 19.3 |  | 0.832 | 0.814 | C | 0.805 | 1.056 |
| 292 | R | None | 45 | 14 | -30.1 | P | 0.448 | 1.506 | C | 0.507 | 1.526 |
| 310 | R | Visual | 5.1 | -80 | 23.1 |  | 0.308 | 0.950 | P | 0.323 | 1.058 |
| 323 | R | Default | 5.9 | 55 | 29.4 | C | 0.505 | 1.287 |  | 0.455 | 0.795 |

***Note:*** The difference in hub role is shown for both groups. Nodes that are not assigned C or P are identified as non-hub. Normalized participation coefficient (PC_norm_), within-module z-score degree (WMZ), connector hub (C), provincial hub (P).
