# Supplementary material for: Altered brain network topology in children with auditory processing disorder: A resting-state multi-echo fMRI study
Source: Neuroimage Clin. 2022 Aug 1;35:103139. doi: 10.1016/j.nicl.2022.103139 (PMC9421544; doi:10.1016/j.nicl.2022.103139)
Supplement: Supplementary data 5 [file mmc5.docx]

**Table S4**

*Results from group differences between HC and APD groups with and without age as a regressor*

| **ROI** | **Label** | **Hem** | **FDR** | **FDR-age** | **Bon** | **Bon-age** | ***p*** | ***P*-age** | **T** | **T-age** |  |
| --- | --- | --- | --- | --- | --- | --- | --- | --- | --- | --- | --- |
| **Gordon parcellation** | | | | | | | | | | |  |
| 331 | Default | R | 0.025 | 0.0333 | 0.023 | 0.0261 | 0.0001 | 0.0001 | 4.0186 | 3.9854 |  |
| **Schaefer parcellation** | | | | | | | | | | |  |
| 56 | | DorsAttn | L | 0.0325 | 0.0375 | 0.0951 | 0.1042 | 0.0003 | 0.0004 | 3.4977 | 3.4708 |
| 93 | | ContA_IPS | L | 0.0364 | 0.0413 | 0.1694 | 0.181 | 0.0008 | 0.0008 | 3.271 | 3.2429 |
| 124 | | DefaultB | L | 0.0364 | 0.0413 | 0.1613 | 0.1727 | 0.0006 | 0.0007 | 3.29 | 3.2629 |
| 147 | | TempPar | L | 0.0325 | 0.0375 | 0.1093 | 0.1196 | 0.0003 | 0.0003 | 3.4459 | 3.4173 |
| 216 | | SalVentAttnA_Ins | R | 0.0364 | 0.0413 | 0.1581 | 0.1622 | 0.0006 | 0.0008 | 3.2989 | 3.29 |
| 294 | | TempPar | R | 0.0325 | 0.0375 | 0.0485 | 0.0541 | 0.0003 | 0.0003 | 3.7427 | 3.7096 |
| 298 | | TempPar | R | 0.0364 | 0.0418 | 0.1243 | 0.1315 | 0.0008 | 0.001 | 3.3963 | 3.3764 |

***Note:*** Age represents results based on age as a nuisance covariate. FDR - false discovery rate,

Bon - Bonferroni correction, *p -* p value, T - test statistic, Hem - hemisphere, ROI - region of interest, Centroid - ROI’s coordinate in standard space, TempOcc – temporal-occipital cortex, TempPar - temporal-parietal network, Ins - insula, DMN - default mode network, SalVentAttn - salience ventral attention network, Cont - control network, DorsAttn - dorsal attention network, L - left hemisphere, R - right hemisphere.
