# Supplementary material for: Altered brain network topology in children with auditory processing disorder: A resting-state multi-echo fMRI study
Source: Neuroimage Clin. 2022 Aug 1;35:103139. doi: 10.1016/j.nicl.2022.103139 (PMC9421544; doi:10.1016/j.nicl.2022.103139)
Supplement: Supplementary data 6 [file mmc6.docx]

**Table S5**

*Overlapping ROIs between Schaefer and Gordon parcellations based on PC measure*

| **Gordon 333** | | | | | | | | | | **Schaefer 300** | | | | | | | | |  |
| --- | --- | --- | --- | --- | --- | --- | --- | --- | --- | --- | --- | --- | --- | --- | --- | --- | --- | --- | --- |
| **ROI** | | **Hem** | | **Label** | **Coordinate** | | | **FDR** | ***p*** | **T** | **ROI** | **Hem** | **Label** | **coordinate** | | | **FDR** | **T** | ***p*** |
| 331 | | R | | Default | 54.4 | 1.1 | -12.9 | 0.0333 | 0.0001 | 3.9854 | 294 | R | TempPar_1 | 54 | 0 | -12 | 0.0375 | 3.7096 | 0.0003 |
| 332 | | R | | VentralAttn | 57.1 | -6.3 | -7.7 | 0.1689 | 0.0013 | 3.0713 | 56 | L | DorsAttnB_TempOcc_1 | -56 | -62 | 2 | 0.0375 | 3.4708 | 0.0004 |
| 89 | | L | | CinguloParietal | -12.7 | -64.9 | 31.8 | 0.1689 | 0.0019 | 2.9524 | 147 | L | TempPar_2 | -56 | -8 | -6 | 0.0375 | 3.4173 | 0.0003 |
| 242 | | R | | VentralAttn | 45.2 | 30.7 | -5.6 | 0.1689 | 0.0024 | 2.922 | 298 | R | TempPar_5 | 58 | -46 | 8 | 0.0418 | 3.3764 | 0.001 |
| 162 | | R | | Default | 12.3 | -51.6 | 34.5 | 0.1689 | 0.0028 | 2.8936 | 216 | R | SalVentAttnA_Ins_1 | 40 | 4 | -12 | 0.0413 | 3.29 | 0.0008 |
| 161 | | L | | VentralAttn | -59 | -18 | -3 | 0.1689 | 0.0036 | 2.8281 | 124 | L | DefaultB_Temp_3 | -52 | -20 | -8 | 0.0413 | 3.2629 | 0.0007 |
| 298 | | R | | Visual | 26.9 | -69.1 | -6.6 | 0.1689 | 0.0034 | 2.7534 | 93 | L | ContA_IPS_4 | -34 | -48 | 46 | 0.0413 | 3.2429 | 0.0008 |
| 1 | | L | | Default | -11.2 | -52.4 | 36.5 | 0.1915 | 0.0055 | 2.627 | 68 | L | SalVentAttnA_Ins_3 | -38 | 0 | 12 | 0.0533 | 3.1051 | 0.0015 |
| 127 | | | L | Default | -53.1 | -11.4 | -16 | 0.1915 | 0.0055 | 2.6254 | 11 | L | VisCent_ExStr_10 | -28 | -84 | 24 | 0.0533 | 3.0996 | 0.0016 |
| 217 | | | R | SMhand | 48.7 | -26.1 | 52.2 | 0.1915 | 0.0057 | 2.599 | 248 | R | ContA_IPS_3 | 40 | -40 | 46 | 0.0555 | 3.0633 | 0.0019 |
| 211 | | | R | DorsalAttn | 38.8 | -42.6 | 40.4 | 0.1953 | 0.0065 | 2.5455 | 114 | L | DefaultA_pCunPCC_1 | -4 | -52 | 16 | 0.0586 | 3.0201 | 0.0022 |
| 290 | | | R | Default | 57.5 | -7.4 | -16.4 | 0.2088 | 0.0076 | 2.5134 | 148 | L | TempPar_3 | -60 | -28 | 2 | 0.0595 | 2.8937 | 0.0027 |
| 120 | | | L | None | -22.5 | 32.1 | -13.6 | 0.2088 | 0.0081 | 2.4611 | 155 | R | VisCent_ExStr_5 | 6 | -88 | -10 | 0.0595 | 2.8922 | 0.0029 |
| 213 | | | R | SMhand | 39.6 | -31.5 | 39.7 | 0.2093 | 0.0088 | 2.4292 | 6 | L | VisCent_ExStr_6 | -46 | -74 | 6 | 0.0595 | 2.8325 | 0.003 |
| 98 | | | L | Visual | -34.2 | -86.6 | -0.5 | 0.2176 | 0.0098 | 2.3898 | 107 | L | ContC_pCun_1 | -10 | -70 | 30 | 0.0595 | 2.816 | 0.0027 |

***Note:*** FDR - false discovery rate, p - p value, T - test statistic value, Hem - hemisphere, ROI - region of interest, coordinate - ROI’s coordinate in standard space, TempOcc - temporalOccipital cortex, TempPar - temporal-parietal network, Ins - insula, IPS - intraparietal sulcus, DMN - default mode network, SalVentAttn - salience ventral attention network, Cont - control network, DorsAttn - dorsal attention network, pCun PCC - precuneus/posterior cingulate cortex, VisCen - visual central, L - left hemisphere, R - right hemisphere.
