# Supplementary material for: Altered brain network topology in children with auditory processing disorder: A resting-state multi-echo fMRI study
Source: Neuroimage Clin. 2022 Aug 1;35:103139. doi: 10.1016/j.nicl.2022.103139 (PMC9421544; doi:10.1016/j.nicl.2022.103139)
Supplement: Supplementary data 7 [file mmc7.docx]

**Table S6**

*Meta-analytic correlation between cognitive terms and significant ROIs obtained from Gordon and Schaefer parcellation*

| **ROI** | **Parcellation** | **Cognitive Label** | **Meta-analytic coactivation (r)** |
| --- | --- | --- | --- |
|  | **Gordon 333** |  |  |
| 331 |  | listening | 0.42 |
|  |  | speech | 0.40 |
|  |  | acoustic | 0.36 |
|  |  | auditory | 0.36 |
|  |  | spoken | 0.36 |
|  |  | sentences | 0.36 |
|  |  | theory mind | 0.36 |
|  |  | comprehension | 0.35 |
|  |  | linguistic | 0.35 |
|  |  | social | 0.35 |
|  |  | speaker | 0.35 |
|  |  | voice | 0.34 |
|  |  | sounds | 0.33 |
|  |  | mind | 0.32 |
|  |  | autobiographical | 0.30 |
|  |  | mental states | 0.30 |
|  |  | speech perception | 0.28 |
|  |  | language | 0.27 |
|  |  | music | 0.26 |
|  |  | vocal | 0.25 |
|  | **Schaefer 300** |  |  |
| 294 |  | listening | 0.39 |
|  |  | theory mind | 0.39 |
|  |  | speech | 0.37 |
|  |  | social | 0.37 |
|  |  | sentences | 0.35 |
|  |  | mind | 0.35 |
|  |  | comprehension | 0.34 |
|  |  | spoken | 0.34 |
|  |  | autobiographical | 0.34 |
|  |  | linguistic | 0.33 |
|  |  | speaker | 0.33 |
|  |  | acoustic | 0.32 |
|  |  | auditory | 0.32 |
|  |  | voice | 0.32 |
|  |  | sounds | 0.3 |
|  |  | mentalizing | 0.28 |
|  |  | language | 0.26 |
|  |  | music | 0.24 |
|  |  | junction | 0.24 |
|  |  | vocal | 0.23 |
| 56 |  | motion | 0.37 |
|  |  | actions | 0.32 |
|  |  | perception | 0.31 |
|  |  | object | 0.25 |
|  |  | gestures | 0.25 |
|  |  | language | 0.25 |
|  |  | visual motion | 0.25 |
|  |  | action observation | 0.24 |
|  |  | lexical | 0.23 |
|  |  | linguistic | 0.23 |
|  |  | verbs | 0.23 |
|  |  | sentence | 0.22 |
|  |  | semantic | 0.22 |
|  |  | words | 0.22 |
|  |  | auditory | 0.21 |
|  |  | comprehension | 0.21 |
|  |  | spoken | 0.21 |
|  |  | visual | 0.20 |
|  |  | audiovisual | 0.20 |
|  |  | listening | 0.20 |
| 93 |  | tasks | 0.67 |
|  |  | working memory | 0.59 |
|  |  | demands | 0.43 |
|  |  | calculation | 0.41 |
|  |  | attention | 0.39 |
|  |  | memory wm | 0.38 |
|  |  | arithmetic | 0.32 |
|  |  | visuospatial | 0.31 |
|  |  | execution | 0.29 |
|  |  | interference | 0.28 |
|  |  | symbolic | 0.28 |
|  |  | maintenance | 0.28 |
|  |  | spatial attention | 0.28 |
|  |  | memory load | 0.26 |
|  |  | phonological | 0.26 |
|  |  | eye movements | 0.26 |
|  |  | task difficulty | 0.26 |
|  |  | action | 0.25 |
|  |  | performance | 0.25 |
|  |  | target | 0.24 |
| 124 |  | comprehension | 0.45 |
|  |  | sentences | 0.45 |
|  |  | language | 0.44 |
|  |  | linguistic | 0.43 |
|  |  | listening | 0.40 |
|  |  | spoken | 0.39 |
|  |  | speech | 0.38 |
|  |  | semantic | 0.37 |
|  |  | auditory | 0.35 |
|  |  | syntactic | 0.35 |
|  |  | speaker | 0.34 |
|  |  | sounds | 0.32 |
|  |  | words | 0.31 |
|  |  | language comprehension | 0.30 |
|  |  | lexical | 0.30 |
|  |  | acoustic | 0.29 |
|  |  | phonological | 0.28 |
|  |  | voice | 0.28 |
|  |  | audiovisual | 0.27 |
|  |  | speech perception | 0.26 |
| 147 |  | listening | 0.73 |
|  |  | spoken | 0.71 |
|  |  | speech | 0.7 |
|  |  | auditory | 0.64 |
|  |  | sounds | 0.63 |
|  |  | linguistic | 0.62 |
|  |  | acoustic | 0.61 |
|  |  | sentences | 0.6 |
|  |  | comprehension | 0.58 |
|  |  | language | 0.58 |
|  |  | voice | 0.56 |
|  |  | music | 0.48 |
|  |  | syntactic | 0.44 |
|  |  | hearing | 0.44 |
|  |  | pitch | 0.42 |
|  |  | lexical | 0.41 |
|  |  | semantic | 0.4 |
|  |  | words | 0.4 |
|  |  | vocal | 0.4 |
|  |  | phonological | 0.39 |
| 216 |  | pleasant | 0.28 |
|  |  | neutral | 0.28 |
|  |  | taste | 0.27 |
|  |  | unpleasant | 0.27 |
|  |  | disgust | 0.27 |
|  |  | pain | 0.26 |
|  |  | emotional | 0.25 |
|  |  | affective | 0.24 |
|  |  | fear | 0.23 |
|  |  | aversive | 0.22 |
|  |  | anxiety | 0.20 |
|  |  | happy | 0.19 |
|  |  | reactivity | 0.19 |
|  |  | valence | 0.19 |
|  |  | arousal | 0.18 |
|  |  | facial expressions | 0.18 |
|  |  | intensity | 0.17 |
|  |  | angry | 0.17 |
|  |  | emotional faces | 0.17 |
|  |  | ptsd | 0.17 |
| 298 |  | facial expression | 0.34 |
|  |  | motion | 0.33 |
|  |  | perception | 0.32 |
|  |  | junction | 0.25 |
|  |  | audiovisual | 0.25 |
|  |  | action observation | 0.22 |
|  |  | gaze | 0.2 |
|  |  | listening | 0.2 |
|  |  | language | 0.18 |
|  |  | linguistic | 0.18 |
|  |  | modality | 0.18 |
|  |  | action | 0.17 |
|  |  | auditory | 0.17 |
|  |  | auditory-visual | 0.17 |
|  |  | speech | 0.17 |
|  |  | sentences | 0.16 |
|  |  | spoken | 0.16 |
|  |  | voice | 0.15 |
|  |  | acoustic | 0.14 |
|  |  | social cognition | 0.14 |

***Note:*** The top 20 associated cognitive terms are shown for each significant ROI according to Gordon and Schaefer parcellations. The meta-analytic terms were derived from the Neurosynth database [(Yarkoni et al., 2011)](https://paperpile.com/c/JO47eU/r2EZ). Cognitive terms were selected based on their meta-analytic coactivation scores (Pearson correlation; uncorrected).
